# The Impact of *NOD2* Genetic Variants on the Gut Mycobiota in Crohn’s Disease Patients in Remission and in Individuals Without Gastrointestinal Inflammation

**DOI:** 10.1093/ecco-jcc/jjaa220

**Published:** 2020-10-29

**Authors:** Andrew Nelson, Christopher J Stewart, Nicholas A Kennedy, John K Lodge, Mark Tremelling, UK IBD Genetics Consortium, Chris S Probert, Miles Parkes, John C Mansfield, Darren L Smith, Georgina L Hold, Charlie W Lees, Simon H Bridge, Christopher A Lamb

**Affiliations:** 1 Faculty of Health and Life Sciences, Northumbria University, Newcastle upon Tyne, UK; 2 Translational and Clinical Research Institute, Newcastle University, Newcastle upon Tyne, UK; 3 IBD Pharmacogenetics Group, University of Exeter, Exeter, UK; 4 Department of Gastroenterology, Royal Devon and Exeter NHS Foundation Trust, Exeter, UK; 5 Department of Gastroenterology, Norfolk and Norwich University Hospitals NHS Foundation Trust, Norwich, UK; 6 Institute of Translational Medicine, University of Liverpool, Liverpool, UK; 7 Department of Gastroenterology, Liverpool University Hospitals NHS Foundation Trust, Liverpool, UK; 8 Department of Gastroenterology, Cambridge University Hospitals NHS Foundation Trust, Cambridge, UK; 9 Department of Gastroenterology, Newcastle upon Tyne Hospitals NHS Foundation Trust, Newcastle upon Tyne, UK; 10 Gastrointestinal Research Group, University of Aberdeen, Aberdeen, UK; 11 Microbiome Research Centre, St George and Sutherland Clinical School, University of New South Wales, Sydney, NSW, Australia; 12 Institute of Genetics and Molecular Medicine, University of Edinburgh, Edinburgh, UK; 13 Edinburgh IBD Unit, Western General Hospital, Edinburgh, UK

**Keywords:** Crohn’s disease, *NOD2* genotype, gut mycobiota

## Abstract

**Background and Aims:**

Historical and emerging data implicate fungi in Crohn’s disease [CD] pathogenesis. However, a causal link between mycobiota, dysregulated immunity, and any impact of *NOD2* variants remains elusive. This study aims to evaluate associations between *NOD2* variants and faecal mycobiota in CD patients and non-CD subjects.

**Methods:**

Faecal samples were obtained from 34 CD patients [18 *NOD2* mutant, 16 *NOD2* wild-type] identified from the UK IBD Genetics Consortium. To avoid confounding influence of mucosal inflammation, CD patients were in clinical remission and had a faecal calprotectin <250 μg/g; 47 non-CD subjects were included as comparator groups, including 22 matched household [four *NOD2* mutant] and 25 non-household subjects with known *NOD2* genotype [14 *NOD2* mutant] identified by the NIHR BioResource Cambridge. Faecal mycobiota composition was determined using internal transcribed spacer 1 [ITS1] sequencing and was compared with 16S rRNA gene sequences and volatile organic compounds.

**Results:**

CD was associated with higher numbers of fungal observed taxonomic units [OTUs] [*p* = 0.033]. Principal coordinates analysis using Jaccard index [*p* = 0.018] and weighted Bray‐Curtis dissimilarities [*p* = 0.01] showed *Candida* spp. clustered closer to CD patients whereas *Cryptococcus* spp. clustered closer to non-CD. In CD, we found higher relative abundance of Ascomycota [*p* = 0.001] and lower relative abundance Basidiomycota [*p* = 0.019] phyla. An inverse relationship was found between bacterial and fungal Shannon diversity in *NOD2* wild-type which was independent of CD [r = -0.349; *p* = 0.029].

**Conclusions:**

This study confirms compositional changes in the gut mycobiota in CD and provides evidence that fungi may play a role in CD pathogenesis. No *NOD2* genotype-specific differences were observed in the faecal mycobiota.

## 1. Introduction

Crohn’s disease [CD] is a chronic relapsing inflammatory disease of the gastrointestinal [GI] tract, which affects the quality of life of over 1.5 million individuals in North America and Europe.^1,2^ Although inflammation may occur at any point along the GI tract, CD commonly affects the terminal ileum and colon^3^ and can lead to significant tissue damage, often necessitating the introduction of biologic therapy, and in many patients surgical intervention.^4–6^ Whereas the aetiology and pathogenesis of CD remain unresolved, the likely mechanisms involve complex interactions between predisposing genes and environmental exposures, which lead to aberrant immune responses against the gut microbiota, resulting in an imbalanced microbial community.^7–10^

CD patients have altered gut bacterial communities that include imbalances in Bacteroidetes, Firmicutes, and Proteobacteria phyla.^11–13^ The gut microbiota of the healthy adult GI tract includes the fungal mycobiota^14–16^ and a study by Sokol and colleagues, which examined the faecal mycobiota in adult CD patients, reported fungal dysbiosis.^17^ Furthermore, a number of studies evaluating the faecal mycobiota in CD report increased prevalence of *Candida* spp.,^17–19^ although the link between increased abundance and disease pathogenesis remains obscure.

The strongest genetic association for CD susceptibility is in the gene encoding the nucleotide-binding oligomerisation domain-containing protein 2 [*NOD2;* also known as CARD15].^20,21^ The three most common CD-associated *NOD2* mutations, R702W and G908R, result in amino acid substitutions and L1007fsinsC results in a premature stop codon and dysfunctional NOD2. In Caucasians, up to 50% of CD patients carry at least one *NOD2* mutation and individuals who carry two mutated *NOD2* alleles have a 20- to 40-fold increased risk of developing CD.^20,21^

NOD2 is a cytosolic pattern recognition receptor that is highly expressed in dendritic cells and macrophages^22^ and Paneth cells,^23,24^ and variably expressed in intestinal epithelial cells.^25^ NOD2 is activated by muramyl dipeptide [MDP], a bacterial cell wall component,^26,27^ and upregulates expression of cytokines, chemokines, and defensins, and triggers adaptive immune responses.^28^ The three major CD-associated mutations are located within the ligand recognition domain of the NOD2 protein and are therefore defective in their ability to sense MDP and trigger autophagy, which impairs gut bacterial handling.^29,30^

A number of studies have investigated the impact of *NOD2*-bacterial interactions in CD,^31–35^ including a retrospective study by Frank *et al*., which found *NOD2*-specific compositional shifts in the intestine-associated bacterial community.^34^ Recently and in contrast, Kennedy and colleagues evaluated the impact of *NOD2* variants on the faecal bacterial community in well-phenotyped CD patients compared with matched controls, and found no *NOD2*-specific alterations in the bacterial communities.^35^

An intriguing study by Wagener and coworkers demonstrated that NOD2 is also activated by the fungal wall component chitin and induces interleukin 10 secretion, so NOD2-dependent recognition of chitin particles dampens inflammatory responses.^36^ Currently, it is unknown what impact *NOD2* genetic variants may have on the gut mycobiota in healthy individuals or patients with CD.

There were three aims of this study. First, we aimed to investigate the faecal mycobiota of CD patients in remission and of non-CD individuals, to determine whether there are unique fungal signatures that distinguish between the two groups, and if *NOD2* genotype impacts upon the composition of the faecal mycobiota. Second, we compared the relative abundance of fungal and bacterial communities in order to identify potential correlations that may be implicated in CD and/or *NOD2* genotype. Third, we aimed to determine whether there are specific correlations between volatile organic compounds [VOC] and bacteria or fungi detectable in the faecal contents.

## 2. Materials and Methods

### 2.1. Study participants

This study used clinical data, stool samples, 16S rRNA gene sequences [accessible from the European Nucleotide Archive, accession number PRJEB21593], and faecal volatile organic compounds [VOC] collected previously for a study to investigate the impact of *NOD2* genotype on faecal bacterial community profiles in CD and non-CD individuals.^35^*NOD2* mutant CD patients were selected if they carried two copies [homozygous or compound heterozygotes] of the CD-associated *NOD2* mutations (R702W [rs2066844], G908R [rs2066845], or L1000fs [rs2066847]). *NOD2* mutant CD patients were matched for age, gender, and geographical location to a wild-type *NOD2* CD patient. To avoid the confounding influence of inflammation, CD patients were confirmed to be in clinical remission as defined by physician assessment and a faecal calprotectin <250 μg/g.^6^ All non-CD subjects had a faecal calprotectin <100 μg/g.^6^ Healthy controls were stratified by the same *NOD2* genotypes, ie *NOD2* wild-type and *NOD2* mutant [compound heterozygotes, other homozygotes, and single heterozygotes]. Two non-CD comparator groups were recruited for this study.^35^ First an environmental control group, where possible a household member [usually an unrelated spouse] of CD participants, was recruited for stool sample collection and saliva for *NOD2* genotyping. Second, a genetic control group of volunteers of known *NOD2* genotype were recruited from the NIHR Cambridge BioResource for stool sample collection. All stool samples were frozen within 24 h of collection. Ethical approval was obtained from the North of Scotland Research Ethics Committee [reference 12/NS/0050]. All study participants provided written consent.

### 2.2. ITS1 sequencing

DNA was extracted from 0.2g stool samples with the DNeasy PowerLyzer PowerSoil kit [QIAGEN, Manchester, UK] and processed according to the manufacturers protocol. A DNA extraction kit negative control was processed alongside each batch of 23 samples and sequenced. ITS1 sequencing was carried out by NU-OMICs [Northumbria University, Newcastle upon Tyne, UK]. Briefly, polymerase chain reaction [PCR] amplification of the internal transcribed spacer 1 [ITS1] region of the eukaryotic ribosomal cluster was amplified using primers ITS1F [CTTGGTCATTTAGAGGAAGTAA] and ITS2 [GCTGCGTTCTTCATCGATGC], using a previously described method,^14^ and was adapted as below. PCR reactions were made up in a total volume of 20 µL using 1x AccuPrime™ Pfx Reaction Mix, 0.5 µM each primer, 500 ng bovine serum albumin, 2.5 U AccuPrime™ Pfx DNA Polymerase, and 14.25 µL of template DNA. PCR cycling conditions were as follows: initial denaturation at 95°C for 2 min, 35 amplification cycles at 95°C for 30 s, 52°C for 30 s, and 68°C for 30 s, followed by a final extension step of 68°C for 7 min. PCR products were cleaned and normalised using the SequalPrep™ Normalisation Plate Kit [Invitrogen, Paisley, UK] and pooled. Pools were quantified using the Qubit™ dsDNA HS Assay Kit [Invitrogen] and diluted to 2 nM. Pools were denatured using sodium hydroxide and diluted to 5 pM, and were sequenced using the MiSeq v3 600-cycle [2 × 300 bp] reagent kit on the Illumina MiSeq platform.

### 2.3. Bioinformatic analysis

Sequences were processed using Mothur [v1.39.5],^37^ and paired end reads were merged using make.contigs with trimoverlap set to true. Any sequence with an ambiguous base was removed from the dataset. Reads were assigned taxonomy using the UNITE database [v8]^38^ and only fungal reads were retained for further analysis. A rarefied observed taxonomic units [OTU] table was generated for downstream analyses of taxa relative abundance and alpha and beta diversity. The total number of raw ITS1 reads was 2 497 751, with a median number of reads per sample of 11 039. After rarefaction, the number of samples employed in subsequent analyses was reduced to 81, with each sample normalised to 1082 ITS1 sequence reads. A total of 90.5% of ITS1 sequences were identified by comparison with the UNITE ITS sequence database. The raw sequence data are available from the European Nucleotide Archive under the study accession number PRJNA607176. Analyses of fungal communities were performed in R [v3.3.1]. To evaluate alpha and beta diversity of fungal and bacterial communities, the number of observed OTUs, Shannon diversity, Jaccard indices, and Bray‐Curtis dissimilarity values were calculated using the ‘Vegan’ package [v 2.5–6].^39^

### 2.4. Volatile organic compounds

The VOC data were generated from a previous study^35^ using a previously described method.^40^

### 2.5. Statistical analysis

Statistical analyses were performed using Prism v.8 [GraphPad, San Diego, USA], Minitab 19 [Minitab, Coventry, UK], or R [v3.3.1]. The distribution of continuous variables (age, body mass index [BMI], and faecal calprotectin) were assessed by Anderson‐Darling normality tests. Proportions of categorical variables were compared using Fisher’s exact test. Significance of non-parametric variables was determined using the Mann–Whitney test for two category comparisons, or the Kruskal–Wallis test when comparing two or more categories, or the Wilcoxon signed rank test for matched pair comparisons. Significance of parametric variables was determined using the two-sample t test for two-category comparisons or paired t tests for matched pair comparisons. Correlation between continuous variables were assessed using non-parametric Spearman rank correlation tests, or parametric variables were assessed using Pearson’s correlation test. Basidiomycota/Ascomycota ratios were calculated for each sample by dividing the percentage relative abundance of Basidiomycota by the percentage relative abundance of Ascomycota. Principal coordinate analysis [PCoA], using unweighted Jaccard or weighted Bray‐Curtis distance, was performed to investigate relationships in β-diversity, and between-variable distances were analysed by PERMANOVA. Where applicable, *p*-values were corrected using the false-discovery rate algorithm [FDR].^41^ To determine the associations between the relative abundance of either fungal genera or bacterial genera and the intensity of stool VOC, we performed sparse partial least squares regression analysis in canonical mode, using MixOmics.^42^

## 3. Results

### 3.1. Characteristics of the study population

**Figure 1.**
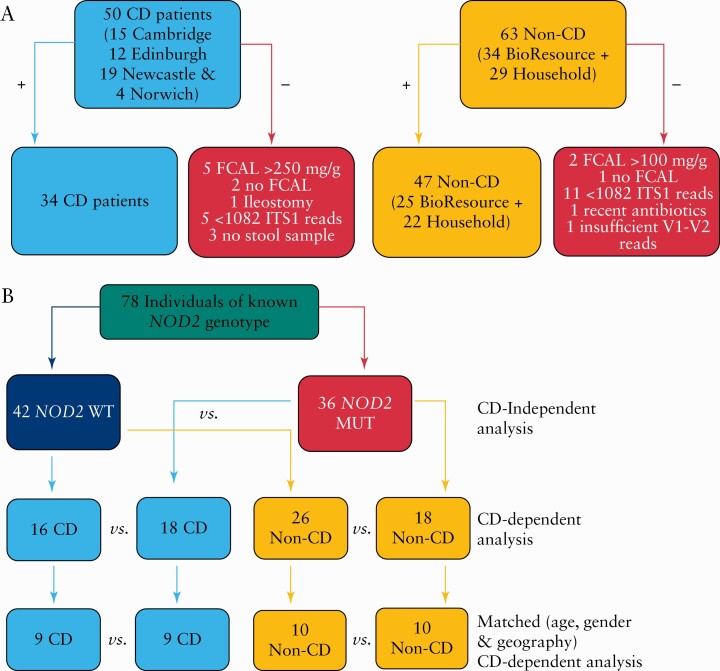
Flow diagrams summarising study participants inclusion criteria and *NOD2* analyses. A] Data from 34 CD patients and 47 non-CD individuals were used in the primary analysis. B] Flow of *NOD2* analyses to evaluate the impact of *NOD2* mutations on the mycobiota independently of disease and to look at disease-*NOD2* specific alterations. CD, Crohn’s disease.

The initial cohort comprised 113 study participants of Caucasian ethnicity [Supplementary Table 1, available as Supplementary data at *ECCO-JCC* online]; 32 samples were excluded from the study and the exclusion criteria are summarised in Figure 1A. A total of 81 participants remained and the characteristics of this cohort are summarised in Table 1A. Three household control subjects could not be *NOD2* genotyped, leaving 78 participants for genotype-stratified analyses [Figure 1B]. There were 34 CD patients [53% *NOD2* mutant] in remission and 47 non-CD individuals [38% *NOD2* mutant] without GI disease [22 household matched volunteers and 25 NIHR Cambridge BioResource volunteers]. CD patients had lower BMIs and higher faecal calprotectin concentrations, and 15% of CD patients were current smokers. A smaller subgroup of 15 CD patients [60% *NOD2* mutant] were matched with their household control [13% *NOD2* mutant]; the characteristics of these groups are summarised in Table 1B. CD patients had lower BMIs and higher concentrations of faecal calprotectin.

**Table 1.** Overview of the clinical characteristics of the study cohort.

A. Crohn’s disease patients and non-Crohn’s disease individuals [*n* = 81]			
Characteristic	Crohn’s disease [*n* = 34]	Non-Crohn’s disease [*n *= 47]	*p*
Gender [F*n*,%]	21 [62%]	22 [47%]	0.259
Age [years], median [Q1-Q3]	53 [44 to 64]	57 [45 to 64]	0.77
BMI [kg/m^2^], median [Q1-Q3]	23.4 [21.4 to 27.0]	25.7 [23.0 to 29.1]	**0.007**
Ethnicity			ND
White—British	32	45	
White—Irish	1	0	
White—Other	1	2	
Faecal calprotectin [µg/g], median [Q1-Q3]	50 [19 to 115]	19 [19 to 30]	**<0.001**
Current smoker, *n* [%]	5 [15%]	0 [0%]	**0.011**
*NOD2* genotype, *n* [%]			ND
Wild-type	16 [47%]	26 [55%]	
Compound heterozygote	11 [32%]	8 [17%]	
Frameshift homozygote	3 [9%]	0 [0%]	
Other homozygote	4 [12%]	6 [13%]	
Heterozygote	0 [0%]	4 [9%]	
Not determined	0 [0%]	3 [6%]	
Antibiotics in past 12 months; *n* [%]	16 [47%]	12 [26%]	0.059
Medication; *n* [%]			ND
NSAID/aspirin	6 [18%]	9 [19%]	
5-ASA	10 [29%]	0 [0%]	
Systemic steroids	1 [3%]	0 [0%]	
Immunomodulator	12 [35%]	0 [0%]	
Anti-TNF	2 [6%]	0 [0%]	
Bile acid sequestrants	9 [26%]	0 [0%]	
Proton pump inhibitors	9 [26%]	3 [6%]	
Probiotics	3 [9%]	3 [6%]	0.692
**B. Crohn’s disease subgroup with household matched individuals *[n* = 30]**			
**Characteristic**	**Crohn’s disease** **[*n* = 15]**	**Household matched individuals [*n* = 15]**	
Gender [F*n*,%]	9 [60%]	6 [40%]	0.466
Age [years], median [Q1-Q3]	44 [38 to 64]	45 [40 to 65]	0.793
BMI [kg/m^2^], median [Q1-Q3]	21.9 [21.1 to 22.9]	26.2 [22.8 to 28.5]	**0.005**
Faecal calprotectin [µg/g], median [Q1-Q3]	50 [19 to 170]	19 [19 to 30]	**0.024**
Current smoker, *n* [%]	2 [13%]	0 [0%]	0.483
Antibiotics in past 12 months, n [%]	7 [47%]	5 [33%]	0.71
Probiotics, *n* [%]	2 [13%]	0 [0%]	0.483
*NOD2* genotype; *n* [%]			ND
Wild-type	6 [40%]	10 [67%]	
Compound heterozygote	3 [20%]	0 [0%]	
Frameshift homozygote	2 [13%]	0 [0%]	
Other homozygote	4 [27%]	0 [0%]	
Heterozygote	0 [0%]	2 [13%]	
Not determined	0 [0%]	3 [20%]	

All data variables were non-parametric and were summarised as the median value [Q1-Q3]. Proportional categorical variables were compared using a 2 × 2 contingency table Fisher’s exact test. The medians of CD and non-CD subjects were compared using the Kruskal‐Wallis test; *p* <0.05 was considered significant and bolded p values show statistically significant differences between groups.

Medications: 5-amino salicylates: mesalazine/sulphasalazine. Systemic steroids: prednisolone. Immunomodulators: azathioprine/methotrexate. Anti-TNF: adalimumab. Proton-pump inhibitor: esomeprazole/omeprazole/lansoprazole/rabiprazole. Bile acid sequestrants: colestyramine/colesevelam.

Non-CD, non-Crohn’s disease; F*n*, female, number; BMI, body mass index; *NOD2*, nucleotide-binding oligomerisation domain-containing protein 2; ND, not determined. 5-ASA, 5-amino salicylates; TNF, tumour necrosis factor; NSAID, non-steroidal anti-inflammatory drug; ND, not done.

### 3.2. Fungal diversity of patients with CD and non-CD individuals

Using the UNITE ITS database, we identified 523 fungal OTUs that were detectable in the stool samples of the cohort, and this identified 184 genera.

**Figure 2.**
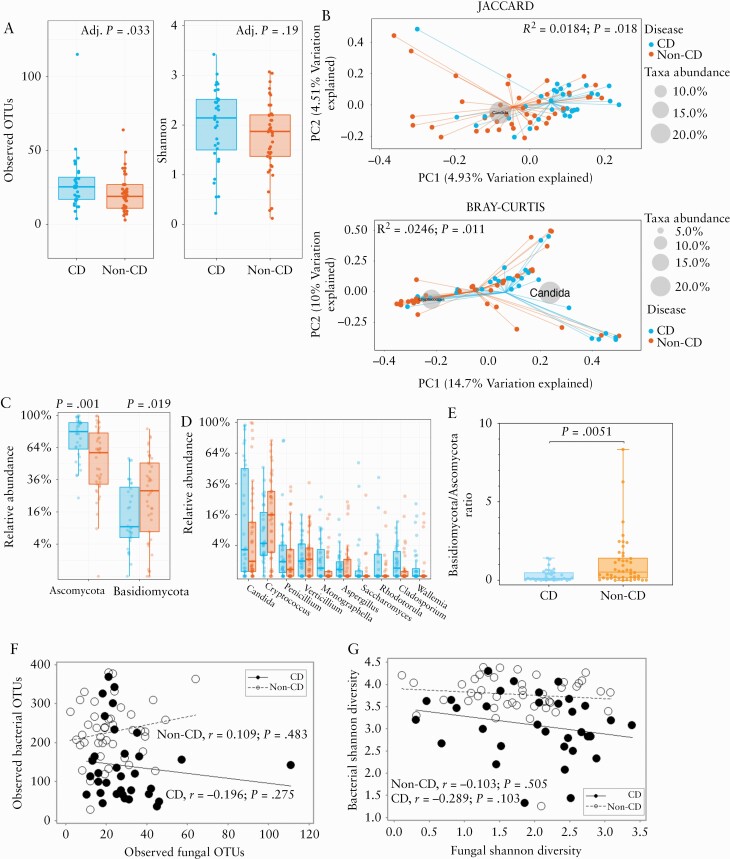
Altered mycobiota diversity in CD patients during remission compared with non-CD individuals. A] Observed number of OTU and Shannon diversity of the mycobiota. The centre line denotes the median, the boxes cover the interquartile range [Q1-Q3], and the whiskers extend to the most extreme data point, which is no more than 1.5 times the length of the box away from the box. Points outside the whiskers represent outlier samples. [The ranges and descriptive statistics are the same for panels C and D] B] Beta-diversity metrics [Jaccard Index and Bray‐Curtis dissimilarity. C] Percentage relative abundance of Ascomycota and Basidiomycota. D] Percentage relative abundance of the top 10 most abundant fungal genera. E] Basidiomycota/Ascomycota ratio. The middle lines are the median ratio, the boxes cover the interquartile ranges [Q1-Q3]. The whiskers show the range from the minimum ratio to the maximum ratio. F] Association between bacterial and fungal observed OTUs in CD and non-CD [Spearman’s rank correlation test applied to both groups]. G] Association between bacterial and fungal Shannon diversity metrics in CD [Pearson’s correlation] and non-CD [Spearman’s rank correlation]. Where relevant, *p*-values were adjusted for multiple comparisons using FDR, and considered significant if *p* <0.05. CD, Crohn’s disease; OTUs, observed taxonomic units; FDR, false-discovery rate.

Comparing the within-sample diversity [alpha-diversity] between CD patients and non-CD subjects, we found a significant difference in observed OTUs [Figure 2A; *p* = 0.033] but there was no difference in Shannon diversity [Figure 2A; *p* = 0.19]. We examined beta-diversity, which considers between-sample variation, of the fungal community using Jaccard indices [considers presence or absence] and Bray‐Curtis dissimilarity [considers relative abundance]. A significant difference was observed in the PCoA using Jaccard indices [Figure 2B; R^2 = ^0.0184, *p* = 0.018] and Bray‐Curtis dissimilarities [Figure 2B; R^2^ = 0.0246, *p* = 0.01; PERMANOVA]. The genus *Candida* and *Cryptococcus* appeared most discriminatory where *Candida* was most associated with CD and *Cryptococcus* was most associated with non-CD. The dominant phyla in the stool mycobiota were Ascomycota and Basidiomycota [Supplementary Table S2, available as Supplementary data at *ECCO-JCC* online]. Ascomycota were significantly more abundant in CD (Figure 2C; false-discovery rate adjusted [FDR-Adj] *p* = 0.001), whereas Basidiomycota were significantly less abundant in CD compared with non-CD [Figure 2C; FDR-Adj *p* = 0.019]. We sought to determine whether there were significant differences in the relative abundance of fungal genera and found no significant difference in genus abundance [Figure 2D]. The Basidiomycota/Ascomycota abundance ratio was found to be significantly lower in CD patients [Figure 2E; *p* = 0.0051]. We investigated whether there were correlations in alpha-diversity between the bacterial and fungal communities, but no significant correlations in the number of observed OTUs [Figure 2F] or Shannon diversity [Figure 2G] by disease status were found.

### 3.3. CD-specific changes in the mycobiota compared with household matched individuals

**Figure 3.**
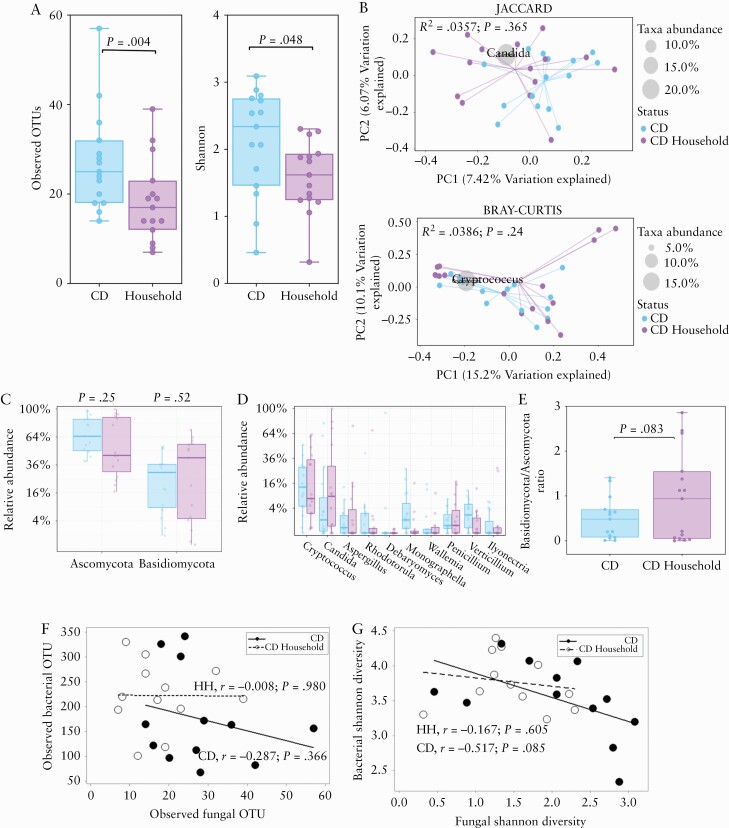
Altered mycobiota diversity in CD patients during remission compared with household matched controls. A] Alpha-diversity metrics [observed OTUs and Shannon]. The centre line denotes the median, the boxes cover the interquartile range [Q1-Q3], and the whiskers extend to the most extreme data point, which is no more than 1.5 times the length of the box away from the box. Points outside the whiskers represent outlier samples. [The ranges and descriptive statistics are the same for panels C and D] B.] Beta-diversity metrics (Jaccard Index [presence/absence of fungal genera] and Bray_Curtis distance [relative abundance of fungal genera]). C] Relative abundance of Ascomycota and Basidiomycota. D] Percentage relative abundance of the top 10 most dominant fungal genera. E] Basidiomycota/Ascomycota ratio. The middle lines are the median ratio, the boxes cover the interquartile ranges [Q1-Q3]. The whiskers show the range from the minimum ratio to the maximum ratio. F] Association between bacterial and fungal OTUs in CD and non-CD [Spearman’s rank correlation test applied to both groups]. G] Association between bacterial and fungal Shannon diversity metrics in CD [Pearson’s correlation] and non-CD [Spearman’s rank correlation]. Where relevant, *p*-values were adjusted for multiple comparisons using FDR and considered significant if *p* <0.05. CD, Crohn’s disease; OTUs, observed taxonomic units; FDR, false-discovery rate.

We sought to compare the fungal mycobiota composition between CD and non-CD shared household contacts. Analysis of the alpha-diversity revealed CD patients had a significantly higher number of OTUs [Figure 3A; *p* = 0.0045, paired t test] and Shannon diversity [Figure 3A; *p* = 0.048, paired t test]. PCoA using Jaccard and Bray‐Curtis indices showed no clustering in the fungal community composition [Figure 3B]. There was no significant difference in the relative abundance of any fungal phyla or genera between CD and household contacts [Figure 3C and D]. The Basidiomycota/Ascomycota abundance ratio was also not significantly different between groups [Figure 3E; *p* = 0.083, Wilcoxon matched pair test]. We investigated whether there were correlations in alpha-diversity metrics between bacterial and fungal communities in CD and household matched subjects, and found no significant correlations in either the number of observed OTUs [Figure 3F] or Shannon diversity [Figure 3G].

### 3.4. Faecal mycobiota-*NOD2* genotype association

**Figure 4.**
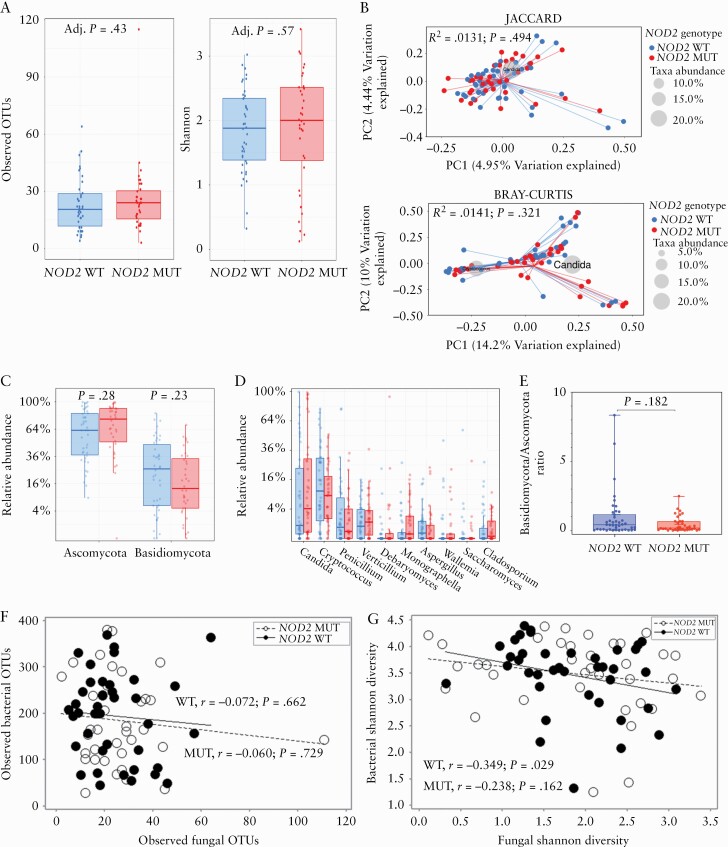
Evaluating the impact of *NOD2* genotype independently of CD. A.] Alpha-diversity metrics [observed OTUs and Shannon] B] Beta-diversity metrics (Jaccard Index [presence/absence of fungal genera] and Bray‐Curtis distance [relative abundance of fungal genera]) C] Relative abundance of Ascomycota and Basidiomycota. The centre line denotes the median, the boxes cover the interquartile range [Q1-Q3], and the whiskers extend to the most extreme data point, which is no more than 1.5 times the length of the box away from the box. Points outside the whiskers represent outlier samples. [The ranges and descriptive statistics are the same for panels C and D]. D] Percentage relative abundance of the top 10 most prevalent fungal genera. E] Basidiomycota/Ascomycota ratio. The middle lines are the median ratio, the boxes cover the interquartile ranges [Q1-Q3]. The whiskers show the range from the minimum ratio to the maximum ratio. F] Association between bacterial and fungal OTUs in *NOD2* wild-type and *NOD2* mutant subjects [Spearman’s rank correlation test applied to both groups]. G] Association between bacterial and fungal Shannon diversity metrics in *NOD2* wild-type subjects [Pearson’s correlation] and *NOD2* mutant subjects [Spearman’s rank correlation]. Where relevant, *p*-values were adjusted for multiple comparisons using FDR and considered significant if *p* <0.05. CD, Crohn’s disease; OTUs, observed taxonomic units; FDR, false-discovery rate.

To examine the impact of *NOD2* genotype on the stool mycobiota, we compared the relative abundance of fungal phyla and genera between 42 *NOD2* wild-type *vs* 36 *NOD2* mutant subjects [19 compound heterozygotes, three frameshift homozygotes, 10 other homozygotes, and four single heterozygotes]. These analyses were independent of Crohn’s disease. Comparing alpha-diversity, we found no significant differences in observed OTUs [Figure 4A; FDR-Adj *p* = 0.43] or Shannon diversity [Figure 4A; FDR-Adj *p* = 0.57]. The fungal profiles were comparable between *NOD2* wild-type and *NOD2* mutant subjects using Jaccard indices [Figure 4B; R^2^ = 0.0131, *p* = 0.494] and Bray‐Curtis [Figure 4B; R^2^ = 0.0141, *p* = 0.321]. There were no significant differences in the relative abundance of Ascomycota [Figure 4C; FDR-Adj *p* = 0.28] or Basidiomycota [Figure 4C; FDR-Adj *p* = 0.23] or genera [Figure 4D].The Basidiomycota/Ascomycota abundance ratio was not found to be significantly different [Figure 4E; *p* = 0.182, Mann‐Whitney test]. We investigated whether there were correlations in alpha-diversity between bacterial and fungal communities and found no significant correlations in either observed OTUs [Figure 4F] in either wild-type *NOD2* [r = -0.072; *p* = 0.662] or mutant *NOD2* [r = -0.060; *p* = 0.729]. However, there was a significant correlation between the bacterial and fungal Shannon diversity [Figure 4G] in wild-type *NOD2* individuals [r = -0.349; *p* = 0.029] but this was not significant in *NOD2* mutant subjects [r = -0.238; *p* = 0.162].

### 3.5. The impact of *NOD2* mutations on the alpha-diversity of the mycobiota in Crohn’s disease

We sought to determine whether *NOD2* was associated with the stool mycobiota in 34 CD patients [16 *NOD2* wild-type *vs* 18 *NOD2* mutant] and 44 non-CD individuals [26 *NOD2* wild-type *vs* 18 *NOD2* mutant]. Three samples could not be conclusively genotyped and so were excluded from this analysis [Supplementary Figure S1A, available as Supplementary data at *ECCO-JCC* online]. There were no significant differences between CD patients with *NOD2* wild-type and *NOD2* mutant in either observed OTUs [FDR-Adj *p* = 0.685] or Shannon diversity [FDR-Adj *p* = 0.85]. In non-CD individuals, we found no significant differences between *NOD2* wild-type and *NOD2* mutant subjects in either observed OTUs [FDR-Adj *p* = 0.435] or Shannon diversity [FDR-Adj *p* = 0.63]. Four non-CD subjects carried a heterozygous *NOD2* mutation. Excluding these samples and repeating the analysis, we found no significant differences between 26 *NOD2* wild-type and 14 mutant subjects in either observed OTUs [FDR-Adj *p* = 0.42] or Shannon diversity [FDR-Adj *p* = 0.80].

Finally, we sought to determine whether *NOD2* would exert the strongest effect on the alpha-diversity of the stool mycobiota in CD patients and non-CD subjects matched for age, gender, and geography. We compared nine CD *NOD2* wild-type subjects *vs* nine CD *NOD2* mutant subjects and found no significant differences in observed OTUs [Supplementary Figure S1B; *p* = 0.436, paired t test] or Shannon diversity [Supplementary Figure S1B; *p* >0.999, Wilcoxon test]. In non-CD individuals we compared 10 *NOD2* wild-type *vs* 10 *NOD2* mutant subjects, and found no significant differences in observed OTUs [Supplementary Figure S1C; *p* = 0.677, paired t test] or Shannon diversity [Supplementary Figure S1C; *p* = 0.544, paired t test].

### 3.6. Fungal and bacterial interactions

**Figure 5.**
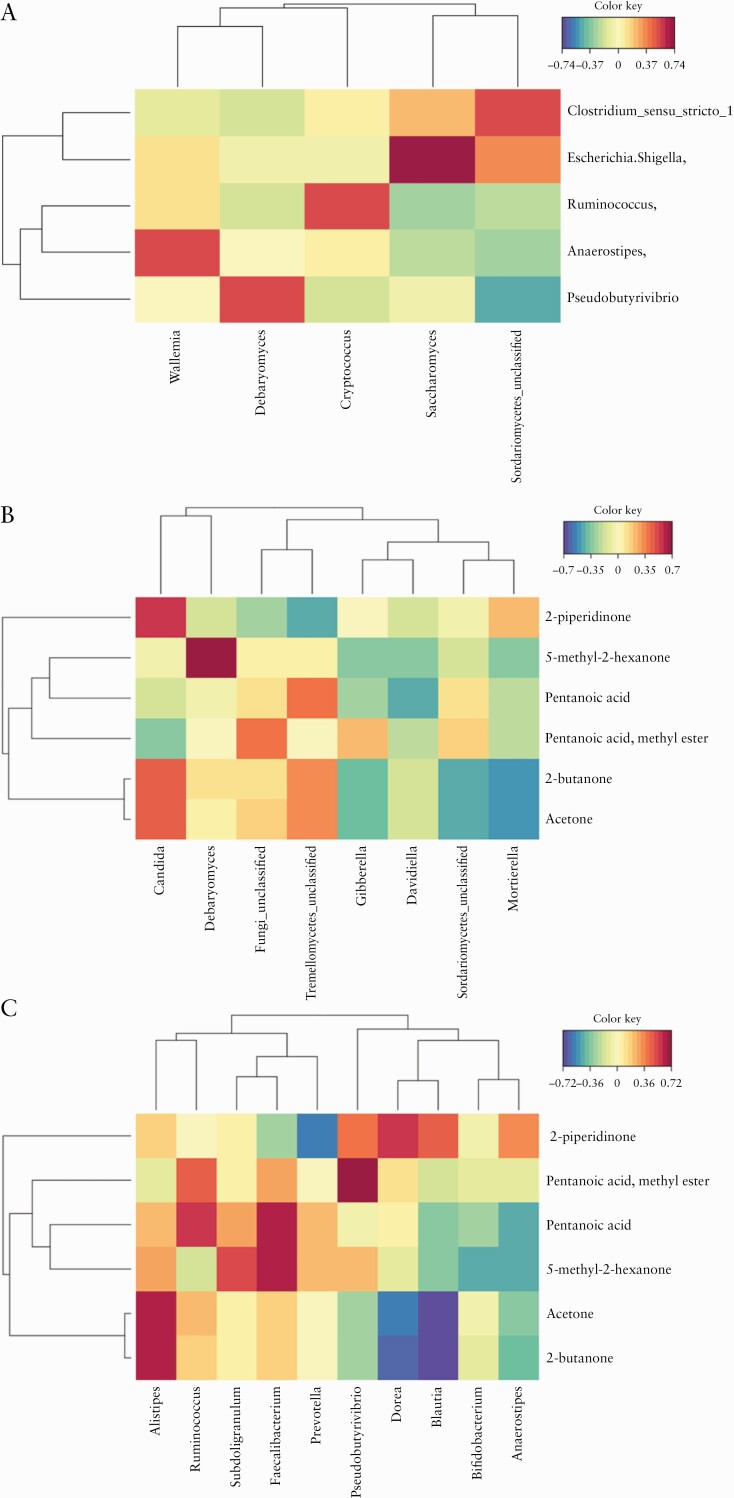
Canonical correlation analyses showing fungal and bacterial interactions and that certain volatile organic compounds [VOCs] associate strongly with the relative abundance of fungal and bacterial communities. These analyses are not stratified by disease or *NOD2* genotype and include *n* = 81 study participants. The red and blue shadings indicate the strength of the positive and negative associations, and yellow shading indicates weak to no association. A] Shows the association between the five most dominant fungal genera with five most dominant bacterial genera. B] Shows the association between eight fungal genera and the six most discriminative VOCs [CD *vs* non-CD]. C] Shows the association between 10 bacterial genera and the six most discriminative VOCs [CD *vs* non-CD]. CD, Crohn’s disease.

To determine whether there were correlations in the relative abundance of fungal and bacterial genera, we performed sparse partial least squared [sPLS] regression. We identified pairwise positive correlations in the faecal mycobiota in samples between genera *Wallemia* and *Anaerostipes, Debaryomyces* and *Pseudobutyrivibrio, Cryptococcus* and *Ruminococcus, Saccharomyces* and *Escherichia/Shigella*, and *Sordariomyces* and *Clostridium-sensu-stricto-1* [Figure 5A]. Previous work on these samples profiled the VOC in the faecal contents of the CD patients and non-CD subjects and found the concentrations of pentanoic acid, 2-butanone, acetone, and 2-hexanone, 5-methyl were significantly lower in CD, and the concentrations of 2-piperidinone and butanoic acid, 3-methyl-, ethyl ester were significantly higher in CD.^35^ We sought to look at the relationship of these CD-discriminating VOCs with the relative abundance of fungal genera, employing a canonical correlation analysis approach [Figure 5B], and identified a strong positive correlation between CD-associated *Candida* spp. and the CD-associated VOC 2-piperidinone, and between *Debaryomyces* and the CD-associated VOC 2-hexanone, 5-methyl-.

We also sought to determine the relationship of the VOCs with the bacterial genera [Figure 5C] and identified strong inverse relationships between *Prevotella* with 2-piperidinone; the bacterial genera *Dorea*, *Blautia*, and *Alistipes* showed a negative correlation with acetone and 2-butanone. The genus *Faecalibacterium* was negatively associated with pentanoic acid and 2-hexanone, 5-methyl-, and *Pseudobutyrivibrio* inversely correlated with the concentration of pentanoic acid.

## 4. Discussion

This study reports that CD patients in clinical remission have higher fungal diversity compared with a non-inflammatory, non-CD comparator group. We found significantly higher observed OTUs in CD, and the fungal profiles of CD patients clustered distinctly from that of non-CD subjects, with *Candida* spp. and *Cryptococcus* spp. found to cluster with CD and non-CD subjects, respectively. We identified shifts in the fungal phyla composition in CD patients, notably lower Basidiomycota that include *Cryptococcus* spp. and higher Ascomycota that include *Candida* spp. However, following correction for multiple comparisons, there were no significant differences found in the relative abundance of fungal genera in CD patients and non-CD individuals. We also report significantly higher alpha-diversity in CD patients compared with their respective household matched control. A strength of this study was that it included well-characterised CD and non-CD participants of known *NOD2* genotype. We compared the faecal mycobiota of subjects stratified by *NOD2* genotype and in the presence or absence of CD, and found that no differences were evident.

We found CD-specific shifts in the abundance of Ascomycota and Basidiomycota. The Basidiomycota/Ascomycota ratio in non-CD subjects was approximately 1:2, and this increased to 1:8 in CD patients. Ascomycota dominated the fungal community in CD patients, and this finding was independent of intestinal inflammation. Our findings confirm previously identified shifts in fungal phyla in the stool mycobiota of CD patients compared with non-CD controls.^17–19,43^ The study by Sokol and colleagues reported that, during intestinal inflammatory flare, the abundance of Basidiomycota increased markedly and the abundance of Ascomycota decreased, and that the Basidiomycota/Ascomycota ratio increased with increasing inflammation.^17^ In contrast, our study excluded any participants with evidence of disease activity, and found that Ascomycota was the dominant phylum in CD patients and that the Basidiomycota/Ascomycota ratio was decreased.

A number of faecal mycobiota studies in CD patients have found an association with *Candida albicans*, although the functional importance of this association remains unknown.^17,19,43^ Our data found that *C. albicans* was the most abundant species in CD patients and also in individuals with CD-associated *NOD2* mutations. A study by Hoarau and colleagues found that *Candida tropicalis* was significantly higher in CD patients and correlated positively with serum anti-*Saccharomyces cerevisiae* antibodies [ASCA].^18^ In our study, *C. tropicalis* was the eighth most abundant species identified in CD patients; however, there were no significant differences found between CD and non-CD subjects. It was also interesting to note that one CD patient was detectable for nearly 80% of all *C. tropicalis* ITS1 reads and this patient had CD-associated *NOD2* mutations.

Recently, Limon and co-authors found that *Malassezia restricta* was more abundant in CD patients with the single nucleotide polymorphism CARD9^S12N^, which normally expresses a key signalling adaptor that plays an important role in anti-fungal immunity.^44^ Curiously, we did not detect *M. restricta* in our cohort. This discrepancy is potentially due to sampling differences. We used stool samples, whereas Limon and colleagues used intestinal wash samples, which have different microbial communities compared with stool.^45^ The observation may also relate to demographic differences of the respective cohorts; all our study participants were recruited from four locations in the UK, whereas the subjects in the Limon study were recruited from California, USA.

Our study found that fungal diversity is higher in CD and this was independent of inflammation. This contrasts with the well-established lower bacterial diversity seen in CD,^11–13,35^ and may indicate that fungi fill this niche in CD. The inclusion of matched VOC data from a previous study^35^ enabled us to look at associations between metabolites and relative bacterial and fungal abundance. One of the primary routes with which the gut microbiota may interact and cross-communicate with each other and the host is through the production of volatile metabolites. Previous studies report that the faecal concentration of the ketone 2-piperidinone is higher in CD during active disease and remission.^35,46^ The present study sought to evaluate correlations between fungal and bacterial communities with faecal VOC, and identified a positive correlation between *Candida* spp. and 2-piperidinone and a negative correlation between 2-piperidinone and *Prevotella* spp., which is intriguing. Future studies to assess if this compound has anti-bacterial properties that confer a competitive advantage on yeast, and enables *Candida* spp. to outcompete commensal bacteria such as *Prevotella*, would be helpful in interrogating the functional relationship between these microbes and VOC.

A study by Wagener and colleagues found that there were chitin-dependent pathways of *NOD2* activation that lead to interleukin-10 secretion and promote intestinal homeostasis,^36^ leading to the hypothesis that the mycobiota of CD patients with *NOD2* mutations would differ from that of wild-type individuals. Our findings suggest that there is no difference in the faecal mycobiota between *NOD2* wild-type or mutant individuals with or without CD. However, CD patients with *NOD2* mutations often have ileal involvement, a more aggressive fistulising and fibrostenotic disease phenotype, and increased risk of postsurgical disease recurrence after ileal resection.^47–49^ Evidence from animal models^50^ and human studies^45^ show that there is a modest correlation between the microbial contents of the stool and ileal wash samples, which suggests that future studies are warranted which should include paired samples to determine the impact of *NOD2* genotype on the ileal-associated microbiome.

The present study has a number of strengths. The study included a cohort of well-characterised CD patients and non-CD subjects with known *NOD2* genotypes. Importantly, the study excluded participants with raised faecal calprotectin levels, thereby reducing the confounding influence of inflammation, which is a key stressor to the gut mycobiota.^17^ The study design enabled comparisons between CD *vs* non-CD controls, CD *NOD2* wild-type *vs* CD *NOD2* mutant, non-CD *NOD2* wild-type *vs* non-CD *NOD2* mutant, and CD *vs* matched household. The latter grouping was important, as unaffected individuals sharing the same household environment with a CD patient may also show signs of bacterial dysbiosis.^51^ Our findings show that the fungal community of CD patients are richer and more diverse than the mycobiota of their respective household controls.

Some study limitations should be noted. Reflective of the complexity of undertaking a genotype-stratified study, despite recruitment from the UK IBD Genetics cohort via several IBD centres, the absolute number of participants and the numbers of participants in each group [CD patients, non-CD subjects, matched household subjects, wild-type *NOD2*, and mutant *NOD2*] and of samples in this multi-omic analysis, are relatively small. Furthermore, this study evaluated the impact of the most common *NOD2* genetic variants on the mycobiota and stratified participants into two groups [wild-type *NOD2* and mutant *NOD2*], as we recognized that it was underpowered to examine the effects of individual genotypes including the effect of rarer *NOD2* variants.^52^ To remove the confounding influence of inflammation, our study recruited CD patients in remission. However, it is possible that the greatest impact of *NOD2* genotype on the gut mycobiota will manifest during active disease. We also did not undertake functional analysis regarding NOD2 activity in the respective CD-associated *NOD2* mutations, and thus did not determine whether there was aberrant fungal sensing or handling in *NOD2* mutant subjects compared with *NOD2* wild-type. Finally, this study characterised fungal taxonomy by employing an ITS1 amplicon DNA sequencing approach, and we recognise that there may be variation in the taxonomic resolution of the fungal communities by targeting alternative genetic markers such as ITS2, 18S, and 28S rRNA.^53^

In conclusion, this study shows that fungal diversity is higher and bacterial diversity is lower in CD. We show CD-specific changes in fungal diversity, which importantly is independent of inflammatory flare. Using stool samples, we could not identify specific associations of *NOD2* genotype with the mycobiota in CD patients or non-CD subjects. *Candida* spp. clustered more closely with CD and highlights the need for further studies to characterise whether *Candida* is a gut commensal or a pathogen in CD patients. Future investigations should also explore the impact of *NOD2* genotype on the ileal-associated mycobiota in larger cohorts stratified by *NOD2* genotype, in order to resolve these complex mucosal interactions.

## Supplementary Material

jjaa220_suppl_Supplementary_Figure_S1
