# Supplementary figures and images for: The Impact of NOD2 Genetic Variants on the Gut Mycobiota in Crohn’s Disease Patients in Remission and in Individuals Without Gastrointestinal Inflammation
Source: J Crohns Colitis. 2020 Oct 29;15(5):800–12. doi: 10.1093/ecco-jcc/jjaa220 (PMC8095387; doi:10.1093/ecco-jcc/jjaa220)

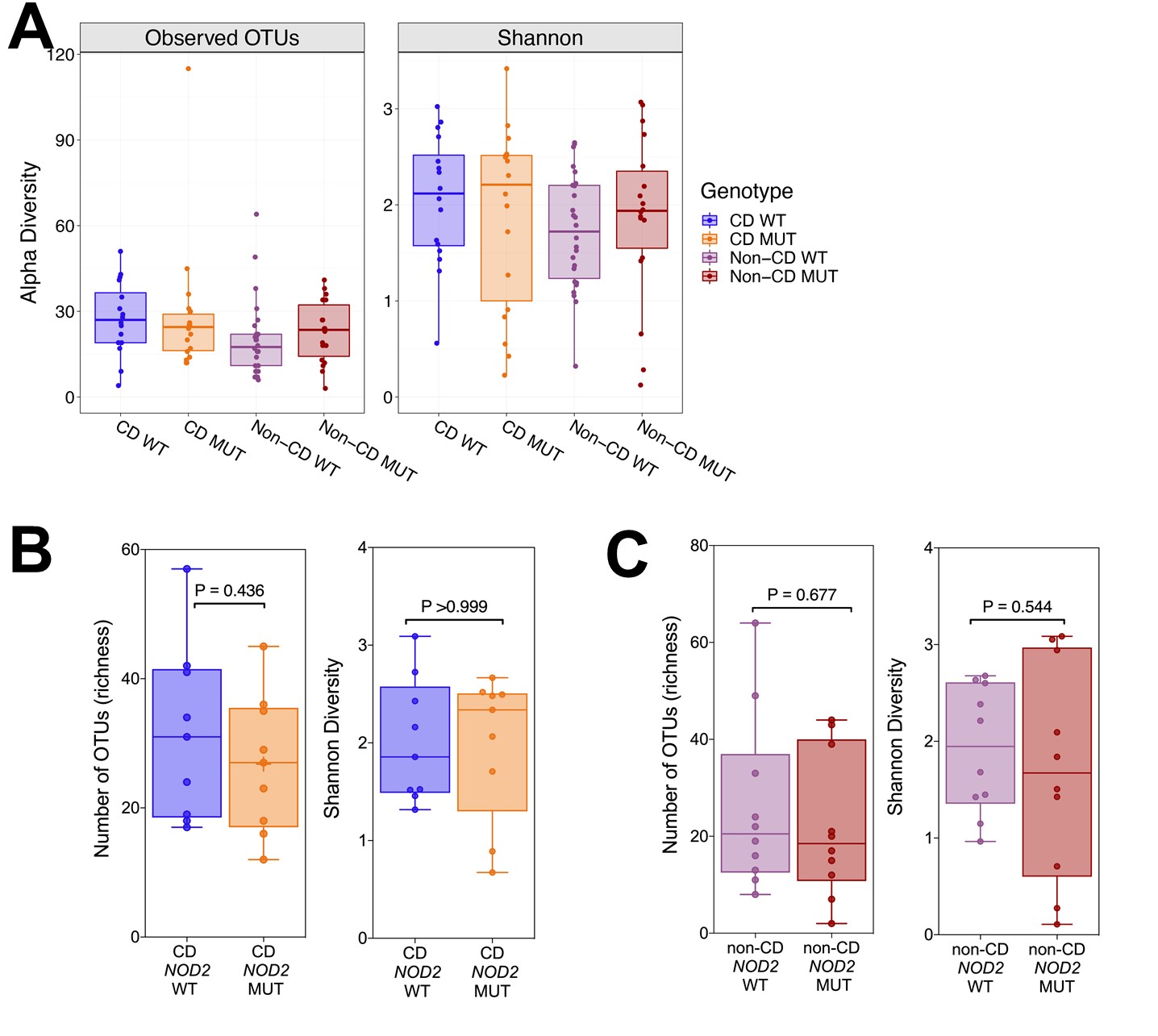

Supplement: jjaa220_suppl_Supplementary_Figure_S1 [file jjaa220_suppl_supplementary_figure_s1.jpeg]
